# Neuro-insights: a systematic review of neuromarketing perspectives across consumer buying stages

**DOI:** 10.3389/fnrgo.2025.1542847

**Published:** 2025-07-11

**Authors:** Raveena Gupta, Anuj Pal Kapoor, Harsh V. Verma

**Affiliations:** ^1^Faculty of Management Studies, University of Delhi, New Delhi, India; ^2^School of Management and Entrepreneurship, Indian Institute of Technology Jodhpur, Jodhpur, Rajasthan, India

**Keywords:** neuromarketing, consumer neuroscience, neuroscientific tools, systematic literature review, consumer buying and decision making process

## Abstract

The application of neurophysiological techniques in marketing and consumer research has seen substantial growth in recent years. This review provides a comprehensive overview of how neuroscience has been integrated into consumer behavior research commonly referred to as “neuromarketing.” While prior reviews have addressed methods, tools, and theoretical foundations, they have largely concentrated on the pre-purchase stage of decision-making. Expanding on this, the current review examines the stage specific affective behavioral and cognitive components neural responses across the full consumer journey. Using the PRISMA framework, the authors systematically analyze stage specific existing neuromarketing literature to present a well-rounded perspective. Moreover, it introduces an integrated framework that aligns neuromarketing insights with each stage of the consumer decision-making process. To support future research, the paper proposes a novel 3 × 3 typology, identifying cross modal interactiona and underexplored areas and gaps in the literature. Overall, this review advances neuromarketing as a rigorous and credible research approach, offering valuable direction for scholars and contributing to its establishment as a recognized discipline within marketing.

## Introduction

Neuromarketing, or consumer neuroscience, is an interdisciplinary field that combines neuroscience, psychology, and economics to explore and influence consumer behavior. Neuromarketing entails analyzing physiological and brain signals to examine the mind, brain, and behavior, aiming to understand, predict, and shape consumer behavior and decision-making (Harrell, 2019). The rise of neuromarketing was fueled by the growing need for more profound insights into consumer behavior, made possible through advancements in neuroscience and technology. Nearly two decades ago, the first wave of neuromarketing literature appeared (Smidts, 2002), and since then, only a few key areas at the intersection of neuromarketing and consumer behavior have been explored—such as decision-making, preferences, choices (Ramsøy et al., 2017), emotional responses, perception, and memory (Ariely and Berns, 2010; Stipp, 2015; Oliveira P. M. et al., 2022). From its initial experiments to broad commercial application, neuromarketing has developed into a vital tool for understanding and shaping consumer decision-making, offering valuable insights into customers' motives, preferences, and choices (Harrell, 2019). As of 2025, the global neuromarketing market is experiencing significant growth, driven by the increasing demand for deeper consumer insights and the adoption of advanced technologies. The market was valued at approximately USD 1.44 billion in 2023 and is projected to reach around USD 3.11 billion by 2032, growing at a compound annual growth rate (CAGR) of 8.9% during the forecast period (Neuromarketing Market Size: Mordor Intelligence, n.d.). This growth is fueled by the rising adoption of neuromarketing across industries such as retail, consumer electronics, and media, as businesses increasingly turn to neuroscience-based methods for deeper consumer insights. North America leads the market, bolstered by substantial investments from major players like Nielsen in advanced neuromarketing technologies. Europe stands out as a key center for academic research, with countries like Spain and Italy at the forefront. At the same time, the Asia-Pacific region is emerging as the fastest-growing market, driven by rising demand in nations such as China and India.

Neuromarketing has gained prominence as a research domain since the early 2000s, marked notably by Montague et al.'s (2004) influential study on the “Neural Correlates of Behavioral Preference for Culturally Familiar Drinks,” popularly known as the “Pepsi vs. Coke” experiment. This foundational work shifted focus toward the non-rational and emotionally driven aspects of decision-making, challenging traditional assumptions rooted in psychology and behavioral science. While neuroscience had begun interfacing with marketing in the early 20^th^ century, it was only in the late 2000s that neuromarketing gained conceptual clarity and commercial traction, helping to legitimize the field (Oliveira P. M. et al., 2022; Morin, 2011). Since then, scholarly and industry interest in neuromarketing has grown rapidly (Karmarkar and Plassmann, 2019; Ramsøy, 2019; Zhang et al., 2021). Given the growth of the field, numerous reviews in neuromarketing employing a systematic literature review approach have been published (Oliveira P. M. et al., 2022; Zhang et al., 2021; Li et al., 2022; Levallois et al., 2021; He et al., 2021). Despite the growing body of neuromarketing research, existing reviews predominantly focus on the behavioral aspects of decision making (Karmarkar and Plassmann, 2019; Ramsøy, 2019), rather than stage specific analysis of neural correlates, creating a gap in our understanding of how consumers' cognitive and affective responses unfold across the full decision-making journey (Ramsøy, 2019). Moreover, the current literature lacks a unified framework (Šola et al., 2022). That integrates neuromarketing insights across distinct decision-making stages, particularly through multimodal approaches combining neurophysiological measures (such as EEG, fMRI, eye-tracking, and GSR) to capture both cognitive processes (e.g., attention, reasoning) and affective dimensions (e.g., emotional arousal, valence). Most existing research emphasizes initial consumer reactions, without adequately addressing how neural and physiological responses contribute to decision reinforcement, post-purchase satisfaction, or brand loyalty (Oliveira P. M. et al., 2022). This gap underscores the pressing need for a stage-specific, multi-method approach in neuromarketing research—one that captures the dynamic interplay of cognition and emotion throughout the entire consumer experience. Addressing this gap forms the foundation of the current research, aiming to advance theoretical understanding and practical application of neuromarketing, especially during the critical purchase and post-purchase phases.

The objective of the present study is to systematically review the literature of neuromarketing based on the cognitive and affective dimensions mapped with stage specific neural correlations, based on the Preferred Reporting Items for Systematic Literature Reviews and Meta-Analyses (PRISMA) framework (Page et al., 2021). In addition, this review aims to (1) establish a conceptual framework that aligns the development of neuromarketing literature with the stages of decision-making, incorporating both cognitive and affective dimensions, and (2) propose a 3 × 3 typology that identifies key research areas across cross-modal methods and decision-making stages. To accomplish these goals, the review seeks to address the following research questions:

RQ1: What are the stage-specific key theories, variables, methodologies, cross modal interactions and neuro-tools commonly used in neuromarketing research?

RQ2: To what extent has neuromarketing research and neural correlates have been explored across the different stages of consumer decision-making?

RQ3: What has been well-studied vs. under-studied at each stage, suggesting future directions, tools, and methods for a more holistic neuromarketing approach.

This review makes five significant contributions to the field of neuromarketing. First, our systematic analysis and synthesis of the literature according to the buying stages of decision making, incorporating stage specific neural correlates. Second, it focusses on empirical studies, commercial applications, and theoretical development, aiming to establish a standardized definition of neuromarketing and resolve its definitional ambiguity. Third, we explore how consumer neuroscience integrates established theories and frameworks to provide insights into cross modal interactions of consumer behavior and synthesize how cognitive vs. emotional processing dominates buying stages (pre-purchase, purchase, and post-purchase). Fourth, the review maps specific neural mechanisms (e.g., reward processing, attention, and emotional arousal) to individual decision stages like problem recognition, information search, evaluation, choice, and post-purchase. Finally, the study proposes a 3 × 3 typology, encompassing decision-making (conscious vs. unconscious vs. both) and buying stages (pre-purchase, purchase, and post-purchase). This typology serves as a roadmap for researchers to explore what is well-studied vs. under-studied under-explored areas in the existing literature (Li et al., 2022; Levallois et al., 2021; He et al., 2021).

This review (Table 1) is valuable from both theoretical and practical viewpoints. Theoretically, it adopts a concept-centric approach to the literature (Webster and Watson, 2002) and examines the intersection of neuromarketing, marketing, and decision-making. The study also highlights the most commonly used neuro-tools and methodologies in the neuromarketing domain. Practically, the review offers valuable insights for product and brand managers, helping marketers better understand consumers' genuine emotional responses to their products and services, rather than relying on potentially biased self-reported data. By exploring the functional capabilities of various tools and their combinations, marketers can optimize campaigns, enhance product design, and develop more effective brand strategies. Ultimately, this review can guide marketers in transitioning to data-driven marketing approaches based on neuroscience.

**Table 1:** Key characteristics of past and present literature reviews on neuromarketing and consumer neuroscience.

**References**	**Aim of the review**	**Adoption of SLR methodology**	**No. of articles**	**Number of journals**	**Interval time**	**Stages of decision making**	**Attitude (A, B, C component captured)**	**Source/databases**	**Classification variables**
González-Morales et al. (2020)	The paper justifies the use of neuromarketing tools in ecological branding strategy by analyzing the literature on branding, ecological branding, and neuromarketing.	NO	61	Unspecified	2015–2019	x	x	Unspecified	•Tools and methods
Pagan et al. (2020)	This paper aims to present research that applied eye tracking and EEG tools to evaluate aspects of sustainability in consumption.	YES	20	Unspecified	2015–2018	x	x	Scopus and WoS	•Tools and methods •Objective
Sung et al. (2020)	To identify new opportunities and challenges for neuromarketing as an applied neuroscience by focusing on neuromarketing papers using 3 or more measures.	NO	Unspecified	Unspecified	2008–2018	x	x	Unspecified	•Tools and Methods •Stimuli
Zuschke (2020)	To understand the roots, current development, and future research avenues of eye-tracking research on consumer decision-making, from the point of view of process-tracing.	Semi-SLR	347	Unspecified	Not explicitly indicated	x	x	WoS	•Tools and methods •Research Themes •Emerging topics and Measurement Indices
Sánchez-Fernández et al. (2021)	The paper studies the current scope, evolution and themes in advertising research	No	203	Unspecified	1986–2019	x	x	WoS	•Tools •Journals •Themes
He et al. (2021)	The paper aims to review consumer neuroscience studies using brain imaging techniques and further propose a framework emphasizing on multi-brain perspective.	Yes	105	Unspecified	2000–2020	Yes	x	WOS, BCI, CSCD, DIIDW, FSTA, KJD, MEDLINE, RSCI, SCIELO	•Tools and methods •Cognitive processes •Applications
Levallois et al. (2021)	To study Neuromarketing from a historical perspective for the development of the field through the examination of public documents available in the online record.	NO	1278	Online records only. NO Journals	2002–2008	x	x	Web	Business history of Neuromarketing
Alsharif et al. (2022)	To study the current advertising research to investigate brain processes pertaining to cognitive, emotional motivational and neurological processes.	Yes	76	Unspecified	2009–2020	x	Yes	WoS	•Brain processes pertaining to emotions, feelings, attention and memory
Krampe (2022).	The paper gives comprehensive guidelines and recommendations concerning mfNIRS in marketing research	NO	13	Unspecified	2009–2021	x	x	Google Scholar, WoS	•Tools and methods •Technicalities of mfNIRS
Li et al. (2022)	The review mainly focuses on the prospects of using EEG in tourism and hospitality research.	NO	34	22	1986–2019	x	x	WoS	•Methods and indices •Themes and theories
Oliveira P. M. et al. (2022)	The paper aims to provide comprehensive knowledge on consumer neuroscience and neuromarketing by clustering and systematizing the complex and dispersed textual information and suggesting the future research agenda.	Yes	469	45	Unspecified	x	x	Scopus	•Tools and methods •Broad themes and Timeline (Yearly Frequency) •Indices and Applications •Future research avenues and Dimensions
Cardoso et al. (2022)	The paper determines the overall performance of neuromarketing research.	Semi- SLR	318	Unspecified	2007–2020	x	x	Scopus	•Topic prominence •Co-authorship and c-word analysis •Productivity measures and impact metrics
Casado-Aranda et al. (2023)	The paper provides an overview of the evolution, current research streams, potential new domains, and theoretical models for neuromarketing research developed in the field of communication.	Yes	861	Unspecified	1979–2022	x	x	WoS	•Citations and Publications •Toolsand methods •Themes and theories
Costa-Feito et al. (2023)	The paper aims to explore the domain of consumer neuroscience and neuromarketing using EEG.	Yes	497	Unspecified	2002–2022	x	x	WoS	•Clustering of marketing research topics employing EEG based on time-period
Landmann (2023)	The paper aims to showcase the current status and use of FaceReader in literature to establish basic guidelines for the overall process of data collection, analysis and interpretation.	Yes	64	43	2013–2023	x	x	EBSCOhost	•Content analysis •Relevance and delimitation •Data Collection, handling and analysis •Goodness and validity
Li et al. (2023a)	The paper discusses the theoretical relevance of neuroscience and its potential themes for tourism domain.	Semi – SLR	9	Unspecified	Unspecified	x	x	EBSCo and Google Scholar	•Tools and methods •Measures of Interest •Dimensions of Emotion
Kakaria et al. (2023a)	The paper aims to review papers using heart rate variability (HRV) and to provide guidelines for the theoretical and practical application of HRV for marketing research.	Yes	33	Unspecified	Till May, 2022	x	Yes	Scopus, WoS, Frontiers, PubMed, Plos ONE, Google Scholar	•Unit of analysis for HRV •Mapping with Marketing mix and SOR model
McInnes et al. (2023)	The paper provides practical and introductory guidelines for the use of EEG in marketing and consumer research.	NO	Unspecified	Unspecified	Unspecified	x	x	Unspecified	•EEG technicalities •Variables of interest
Rodríguez et al. (2023)	The paper aims to systematically review the study of packaging and branding from the consumer neuroscience viewpoint.	Yes	258	Unspecified	2016–2020	x	x	WoS	•Neural coordinates •Cognitive processes •Tools and methods •Citations and co-occurrence clusters
Zhang et al. (2023)	The paper provides an overview of neuromarketing techniques and methods contributing to marketing and consumer research	NO	Unspecified	Unspecified	2016–2021	x	Yes	WoS	•Tools and methods •Context and Theory •Indices, Key Themes and Application
Alzboun et al. (2024)	The paper aims to highlight potential of neuro-tourism as a discipline and underscore its importance in tourism industry	Yes	237	Unspecified	2006–2024	x	x	WoS and Scopus	•Neuro-tourism topics and evolution •Strustural topic modeling •Topic- congruence •Network analysis
Srivastava and Bag (2024)	The paper explores the potential for face recognition and neuromarketing in modern day marketing	Yes	20	Unspecified	Unspecified	x	x	Scopus	•Theory, Context and Methodology •Themes •Antecedents, decision and outcome
Wang et al. (2024)	The paper presents bibliometric and systematic analysis of EEG studies in the realms of consumer behavior	Yes	53		2005–2021	x	x	WoS	•Keywords and co-words analysis •Thematic Structure Analysis
Cenizo (2025)	The paper examines the application of neuromarketing to web environment	Yes	40	Unspecified	2006–2024	x	x	Scopus and WoS	•TCCM
Song et al. (2025)	The paper aims to provide a comprehensive understanding of neuromarketing, its historical development, methods and instruments along with ethical consideration.	Semi-SLR	Unspecified	Unspecified	2000–2024	x	x	Scopus, PubMed and Google Scholar	•Evolution of Neuromarketing •Pros and Cons •Tools and methods •Ethical Consideration
**This Review**	**This review provides an overview of the most relevant theories, variables, methodologies, and tools applied in neuromarketing research in relation to the stages of consumer decision-making and proposes a comprehensive framework for future researchers**.	**Yes**	**109 Union of ABS, Scimago and ABDC journal (Business, Management, Engineering, Social Science, Tourism)**	**55**	**2020**–**2025**	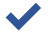	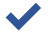	**Scopus, WoS and EBSCO**	•**3** **×3 Typology and Cross Listings** •**Theories mapped with tools, methods and attitudes** •**Capabilities of neuro-metric and non-neuro-metric to measure the variables** •**Neuro-marketing framework and attitudinal responses mapping** •**Alignment with Buying decision phases**

### Rational of the study

The roots of neuromarketing, which emerged in the early 2000s, can be traced back to foundational psychological theories of perception, learning, and motivation (Watson, 1913). During this time, classical conditioning in advertising proposed that emotions linked to advertisements could shape consumer behavior. This concept laid the foundation for the notion that marketing could subconsciously influence behavior through emotional appeal. By the mid-20^th^ century, cognitive psychology began to challenge behaviorism's exclusive emphasis on observable behavior, highlighting instead the importance of mental processes in decision-making (Simon, 1957). This shift in research focus moved the emphasis from external stimuli to a deeper understanding of the cognitive mechanisms driving consumer choices. This research approach contributed to a shift in focus from solely external stimuli to a more comprehensive understanding of the cognitive, affective, and behavioral processes underlying consumer decisions. In the late 20^th^ century, the advent of neuroimaging techniques like EEG (Electroencephalography) enabled researchers to directly observe brain activity in response to marketing stimuli. These tools provided a scientific foundation for exploring emotional and cognitive responses to advertisements, brands, and products. This signaled a theoretical shift from psychological models focused on observable behavior to neuroscientific approaches that explored the brain's internal processes (Raichle et al., 2001). In the early 2000s, the term neuromarketing was introduced, marking the beginning of companies using neuroscientific techniques to gain insights into consumer behavior (Montague et al., 2006). Neuromarketing was further shaped by Daniel Kahneman's dual-process theory of decision-making, introduced in 2011. He identified two modes of thinking: System 1, which is fast, automatic, and emotional, and System 2, which is slow, deliberate, and rational. Kahneman emphasized that System 1, driven by emotions and intuition, plays a key role in effective marketing, as it taps into the subconscious and emotional responses of consumers. The rise of neuromarketing represents the culmination of decades of research spanning psychology, neuroscience, and economics (Glimcher and Rustichini, 2004). However, between 2002 and 2020, evolving definitions and emerging concepts led to a degree of ambiguity surrounding the term. Moreover, decision-making is a multifaceted cognitive process that involves the integration of emotional and behavioral elements across distinct stages. To fully grasp how individuals move through the five phases of decision-making, problem recognition, information search, evaluation of alternatives, selection, and post-choice evaluation, a comprehensive, multidimensional perspective is essential. Conventional models often fail to account for the intricate interactions among emotion, cognition, and behavior. This study aims to address that limitation by examining the emotional and behavioral dynamics at each stage, and how these are influenced by cross-modal interactions captured through neurometric techniques.

Furthermore, existing neuromarketing research has largely concentrated on the pre-purchase phase, examining how the brain reacts to marketing stimuli prior to a buying decision. In contrast, there has been limited exploration of its application during the purchase and post-purchase phases—stages where consumer satisfaction, brand loyalty, and advocacy may be significantly influenced by neuropsychological factors (Lin et al., 2018). In addition, how do the affective, behavioral and cognitive responds across the stages has been largely unexplored. This study proposes a cross-modal methodology, integrating multiple neurometric tools to enrich data interpretation and enhance methodological rigor. By aligning physiological and neural indicators with behavioral observations and self-reports, we aim to reveal how emotional states interact with decision strategies throughout each phase. This multimodal approach also compensates for the limitations inherent in any single measurement technique, providing a more complete picture of the underlying mechanisms. This broader perspective can support the development of robust theoretical models and practical strategies, offering deeper insights into how emotional, cognitive, and neural processes shape decision-making throughout the entire consumer journey.

Analyzing all five stages mapped to the affective, behavioral and cognitive components provides a comprehensive view of decision-making, especially since these phases do not always follow a linear progression (Lemon and Verhoef, 2016). It is essential to account for their occurrence and specify the continuum between conscious and unconscious decision-making. Marketing literature often focuses on proxies for the pre-purchase, purchase, and post-purchase phases, leading to fragmented and subjective insights into these stages (Kotler et al., 2015). A deeper review of literature at the intersection of marketing and consumer neuroscience, within the context of consumer decision-making stages, offers a more accurate understanding of actual consumer behavior rather than relying on proxy estimates of decisions. From the preceding discussion, it can be concluded that neuromarketing has evolved considerably over time, is inherently dynamic, and remains a highly pertinent area of research. In particular, aligning the literature on neuromarketing based on the cognitive and affective dimensions mapped with stage specific neural correlations is increasingly important, as it enables a more thorough and organized understanding of how neurological and psychological factors shape consumer behavior throughout the decision process. By connecting neuromarketing insights to these stages, researchers can identify specific neuro and non-neuro metric tools which may be used to identify specific neural and emotional triggers that influence consumer choices at each phase.

## Systematic review approach

In this section, the methodology adopted for carrying out the systematic literature review process is elaborated.

### Methods

To collect data, we utilized the Preferred Reporting Items for Systematic Reviews and Meta-Analyses (PRISMA) framework (Page et al., 2021), a widely recognized method commonly applied in marketing-related systematic reviews and meta-analyses (Paul and Barari, 2022). This structured approach provides a transparent and methodical process for conducting and reporting reviews, promoting thoroughness, replicability, improved reporting quality, consistency, and minimizing bias in the analyzed studies.

#### Search strategy

Due to the interdisciplinary nature of the research, we explored EBSCOhost Business Source Complete, Scopus, and Web of Science to ensure broad coverage across relevant studies. Specifically, for this review, a search was conducted on April 14, 2025, focusing on neuromarketing, consumer neuroscience and consumer decision making. This initial query yielded 3,203 potentially relevant articles. Appendix A outlines the keyword search strategy along with the predefined inclusion and exclusion criteria used to guide the subsequent stages of the review.

#### Screening procedures

The identified articles were imported into the citation management software EndNote X9, where the initial pool of 3,203 studies was refined to 338 using the Find Duplicates function. Next, exclusion criteria were applied to filter out publications unrelated to “business,” “management,” “accounting,” “engineering,” or “social science.” Articles not published in academic journals, those outside the 2020–2025 timeframe, or written in languages other than English were also excluded. As part of the inclusion criteria, only documents classified as articles or reviews were retained, with a particular emphasis on source titles relevant to the business and management fields.

#### Record eligibility

**Figure 1:**
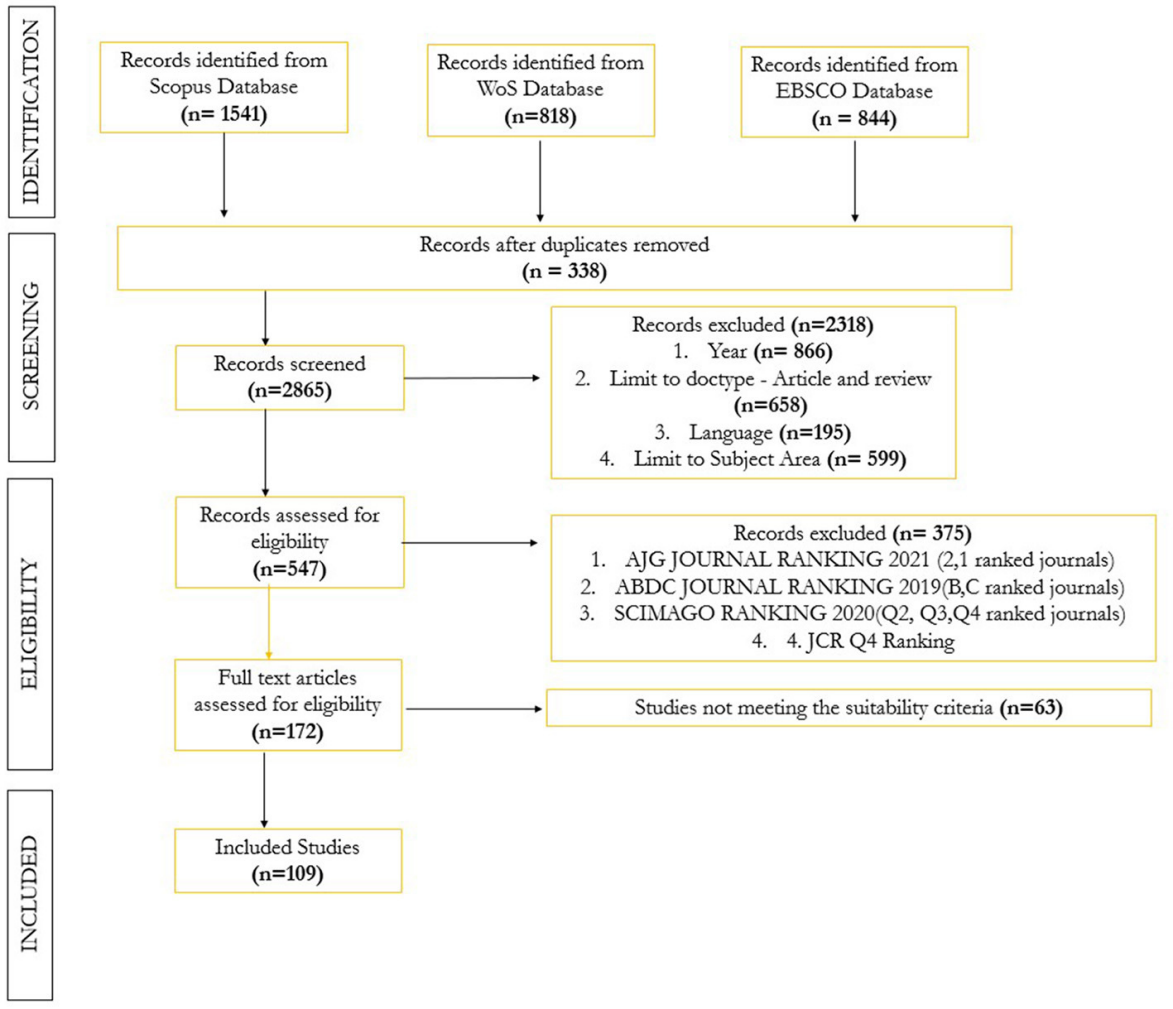
PRISMA flowchart.

The field of neuromarketing and consumer neuroscience is highly diverse and fragmented—not only in terms of the topics and areas explored, but also in the methodologies employed, contexts studied, and application approaches—resulting in a heterogeneous body of literature varying widely in both subject matter and quality (Ramsøy, 2019; Zhang and Lee, 2022). To ensure the inclusion of high-quality, peer-reviewed research and to generate credible, broadly accepted insights, we conducted a quality screening of our initial sample. Following Zuschke (2020), we prioritized studies published in high-ranking journals, including those rated as Grades 3, 4, or 4^*^ in the Chartered Association of Business Schools (CABS) list (Baldacchino et al., 2015), A or A^*^ in the ABDC journal list, or classified as Q1 in Scimago. In addition, Journal Citation Reports (JCR) Q4 were excluded. Given the interdisciplinary nature of neuromarketing and consumer neuroscience, we adopted a combination of these journal ranking criteria, in line with recommendations from prior review studies (Soundararajan et al., 2018). As a result of this filtering process, 172 research articles were shortlisted for further evaluation. These 172 studies were then assessed using fit-for-purpose criteria, which evaluate whether research aligns with the specific objectives of a review, as outlined by Boaz and Ashby (2003). For this review, studies were retained if they met the following conditions: (1) a clear link between consumer neuroscience and marketing, business, or management; (2) a focus on consumer decision-making; (3) relevance to stages of the purchasing process; and (4) inclusion of attitudinal factors. Full-text analysis was conducted to determine alignment with the core research question. This final screening resulted in a refined sample of 109 studies (Figure 1).

## Proposed conceptual framework

**Figure 2:**
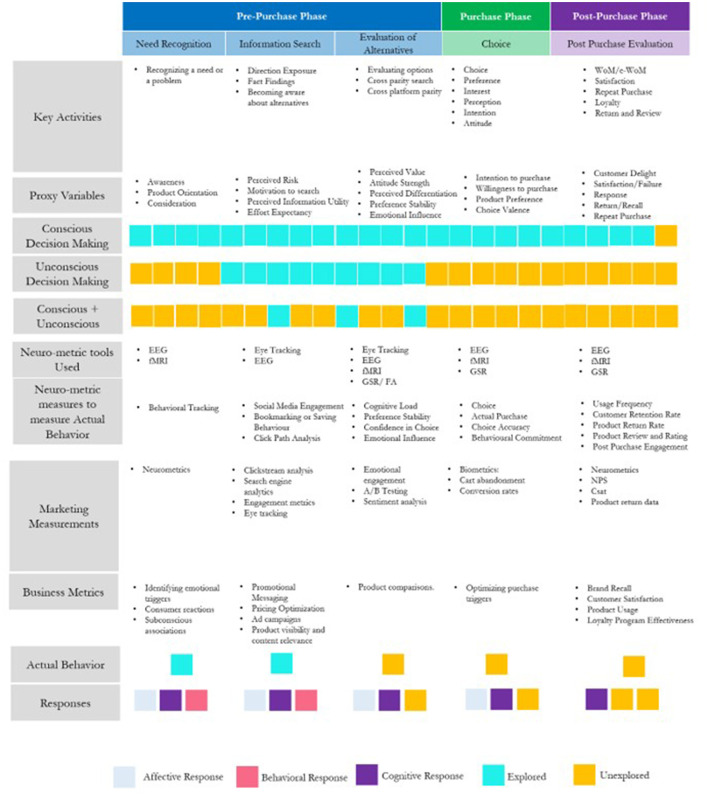
Proposed conceptual framework.

This section presents the conceptual framework guiding our review. As illustrated in Figure 2, the framework maps the various stages of consumer decision-making to attitudinal, behavioral, and cognitive components, emphasizing the key activities associated with each stage. Consumer purchase decision-making is typically described in five distinct stages: (1) Need recognition, (2) seeking information, (3) assessing alternatives, (4) making the purchase, and (5) participating in post-purchase activities (Yadav et al., 2013). These stages can also be categorized into three general phases: pre-purchase, purchase, and post-purchase (Schiffman et al., 2011). A cross-model interaction of all neuro as well as non-neuro-metric tools also highlights the extend of usage of these tools across a customer's journey mapping.

### Need recognition stage

The pre-purchase stage encompasses all activities a consumer undertakes prior to making a purchase, including need recognition, information gathering, and evaluating available alternatives (Lemon and Verhoef, 2016). Need recognition, a fundamental aspect of the pre-purchase stage, occurs when a consumer becomes aware of a problem or desire, which may arise from internal stimuli (e.g., hunger or thirst) or be triggered by external influences such as advertisements or social interactions (Sung et al., 2020; Yun et al., 2021). During this phase, consumers begin to form awareness and interest in particular brands, products, or shopping environments. Researchers often use proxies like perceived convenience and enjoyment to evaluate consumer motivations at this stage (Singh and Swait, 2017). Neuromarketing has contributed significantly to understanding need recognition by uncovering the neural mechanisms involved. For example, studies have shown that areas like the striatum—linked to the brain's reward system—are activated in anticipation of fulfilling a recognized need. Techniques such as EEG and fMRI help reveal how emotional and cognitive processes shape early brand perceptions, enabling marketers to craft stimuli that connect with consumers on a subconscious level. These findings highlight the importance of tailoring marketing strategies to both the psychological and social aspects of the pre-purchase experience (Venkatraman et al., 2015; Yun et al., 2021), as various brain regions are engaged—from those involved in basic emotional reactions to areas responsible for more complex cognitive associations. Venkatraman et al. (2015), using fMRI, demonstrated that the ventral striatum—a key part of the mesolimbic system linked to reward processing and motivation (Tremblay et al., 2009)—exhibits significant activation in response to specific marketing cues. For instance, Linder et al. (2010) found that food labeled as “organic” triggered stronger activity in the ventral striatum compared to food labeled conventionally, underscoring how labeling can influence consumer perception at a neural level. These findings underscore the critical role of reward anticipation in shaping consumer preferences during the pre-purchase phase. Neuromarketing tools such as functional magnetic resonance imaging (fMRI), electroencephalography (EEG), and eye-tracking have significantly enhanced our understanding of how consumers react to marketing stimuli at this stage (Harrell, 2019). fMRI research indicates that brand recognition activates brain regions associated with emotion and memory, which can influence product preferences even before conscious evaluation occurs. Similarly, EEG studies demonstrate that emotional and visual cues effectively capture attention and enhance memorability—both essential elements for pre-purchase consideration (Harrell, 2019). Additional studies employing brain imaging and physiological measures have explored need recognition in greater depth, revealing that stimuli often trigger activation in the ventral striatum, linked to reward anticipation, and the amygdala, which processes emotional relevance (Lee et al., 2007; Knutson et al., 2007; Berns and Moore, 2012; Vezich et al., 2017; Casado-Aranda et al., 2018). Collectively, this body of research suggests that neural responses during the need recognition stage play a pivotal role in shaping consumer behavior and decision-making.

### Exploration of information and assessment of alternatives stage

In the pre-purchase phase, consumers actively seek information and evaluate various options, which may involve comparing multiple products or concentrating on a particular one (Lee et al., 2017; Yadav et al., 2013). During this process, they assess how well each option aligns with their needs and preferences, while also considering factors such as cost, time, and effort. If the perceived effort exceeds the anticipated benefits, consumers may choose not to proceed with the purchase. Researchers often use metrics such as cognitive effort, attention, search behavior, and product consideration to assess consumer intent at this stage (Zhu et al., 2020; Melumad and Meyer, 2020; Ghose et al., 2013; Goldstein and Hajaj, 2022; Raphaeli et al., 2017; De Haan et al., 2018; Kukar-Kinney et al., 2022; Zhang et al., 2021). However, Liu et al. (2023) contends that these measures largely indicate behavioral intent rather than actual consumer behavior. For example, attentional focus—commonly evaluated using Likert scales—may not reliably reflect true attention levels, as such subjective assessments are difficult to validate through self-reported surveys (Melumad and Meyer, 2020). Neuromarketing offers valuable insights into the information search and evaluation stages by analyzing both neural and behavioral responses. Research has shown that visual factors, such as product placement and advertisement design, significantly impact how consumers process and prioritize information. EEG and fMRI studies indicate increased activity in the prefrontal cortex during logical assessments, while the amygdala becomes more active in response to emotional elements related to branding or perceived product value. Moreover, reward-related neural activity in the striatum plays a key role during the evaluation phase, as consumers integrate emotional and cognitive inputs—such as pricing and perceived benefits—when comparing alternatives (O'Reilly et al., 2013). EEG studies also detect event-related potentials (ERPs), such as the P300 and late positive potential (LPP), which are indicators of cognitive and emotional processing during decision-making (Polich, 2007; Hajcak and Foti, 2008). These insights deepen our understanding of how the brain processes information, enabling the development of more effective content that resonates with the cognitive and emotional dynamics involved in the information search stage.

### Buying decision phase

In the purchase decision phase, consumers make several critical choices, including whether to proceed with the purchase, which product(s) to select, how much to buy, which retailer to choose, and the best time to make the purchase. Other factors, such as delivery times for online orders, also influence decisions in this stage (Lee et al., 2017; Yadav et al., 2013). Consumers assess aspects like waiting times as part of their evaluation process (Meißner et al., 2020; Shen et al., 2016), payment options (Boden et al., 2020; Liu and Dewitte, 2021), and their willingness to pay (Garg and Lerner, 2013; Herhausen et al., 2019; Kaatz et al., 2019). However, these variables often fail to capture actual purchase intent (Karmarkar et al., 2021; Plassmann et al., 2012), with a few exceptions found in studies using real-time data from third-party platforms. Despite the growing interest in neuromarketing, the purchase stage remains underexplored compared to earlier stages like information search and evaluation (Yun et al., 2021). Most research has focused on pre-purchase factors like branding, advertising, and packaging design, often neglecting the immediate and dynamic cognitive and emotional responses that arise at the point of sale. This gap is primarily due to the challenges of capturing real-time neural and behavioral data during actual purchasing moments. The complexity of measuring processes like shifts in attention, emotional arousal, and decision-making at the moment of purchase has made it difficult for researchers to fully understand the true triggers and motivations behind consumer behavior during this critical phase (Plassmann et al., 2012; Meißner et al., 2020). As a result, the purchase stage remains less explored compared to earlier stages like information search and evaluation (Karmarkar et al., 2021 and Yun et al., 2021). The complexity of studying the purchase stage arises from the difficulty of replicating realistic shopping environments while utilizing tools like fMRI or EEG. Laboratory settings often suffer from low ecological validity (Ariely and Berns, 2010), making it challenging to capture the immediate pressures and contextual influences that shape decision-making in real-world retail situations. Consequently, experiments conducted in controlled environments may fail to accurately reflect the dynamic nature of in-the-moment consumer choices, which are impacted by factors such as time constraints, social cues, and environmental stimuli (Plassmann et al., 2012; Meißner et al., 2020). This limitation hinders the ability to fully replicate the decision-making processes consumers undergo during actual purchases (Zhang et al., 2021). Studies have highlighted promising approaches, such as using portable neuroimaging tools to examine decision-making in more naturalistic settings, although these efforts are still limited in scope. For example, Herrando et al. (2022) demonstrated that online customer reviews can unconsciously trigger arousal and pleasure, leading to a purchase. Addressing this gap is crucial, as the purchase stage is where consumer intentions are translated into actual behavior, offering businesses vital insights into customer preferences and choices (Karmarkar et al., 2021; Plassmann et al., 2012). The ability to capture real-time neural responses and behaviors during the purchase decision process could deepen our understanding of what drives actual purchases, providing valuable data to refine marketing strategies and optimize the consumer experience (Meißner et al., 2020; Plassmann et al., 2012).

### Post-purchase decision stage

After purchasing a product, consumers often assess their experience in comparison to their initial expectations (Lee et al., 2017; Yadav et al., 2013). They evaluate the product's strengths and weaknesses, which can influence their decision to recommend it or express dissatisfaction. This evaluation typically involves providing feedback through word-of-mouth, social media, or personal conversations. Consumers may also emphasize the positive aspects of the product to rationalize their purchase. This post-purchase evaluation affects their overall satisfaction, likelihood of repurchasing, and potential future service requests or returns. Post-purchase intent is often measured using proxy variables, while feedback from returns and service requests provides valuable insights into consumer behavior. This feedback is particularly useful for actual users, as opposed to those who only considered making a purchase.

In neuromarketing, post-purchase behavior is examined by analyzing how consumers evaluate their purchase afterward, with a focus on emotional and cognitive responses. For example, Hamelin et al. (2020) and Grigaliūnaitè and Pilelienè (2017) explored how storytelling leads to a more immediate shift in decision-making during the post-purchase stage of the consumer life cycle. However, the post-purchase phase remains relatively underexplored in neuromarketing, as most research tends to concentrate on earlier stages of the consumer journey. This gap is partly attributed to the challenges of capturing neural and physiological responses related to post-purchase emotions such as satisfaction, loyalty, or regret. These emotions develop over time and are often difficult to measure in controlled environments (Lee et al., 2017; Plassmann et al., 2012). Additionally, research limitations arise from a focus on immediate consumer reactions, rather than on long-term behavioral and emotional outcomes (Garczarek-Bak et al., 2021; Cakir et al., 2018; Lee et al., 2017). Addressing these gaps is essential, as the post-purchase stage provides valuable insights into the effectiveness of marketing strategies and consumer satisfaction (Lee et al., 2017; Zhang and Lee, 2022). Neuroimaging studies, such as those using fMRI, reveal that brain regions like the ventral striatum and prefrontal cortex play a role in processing post-purchase satisfaction and dissonance (Knutson et al., 2007). The ventral striatum is associated with the reward and pleasure derived from the purchase (Delgado et al., 2000), while the prefrontal cortex helps assess the value and fulfillment of the decision. EEG studies capture event-related potentials (ERPs), such as error-related negativity (ERN), which can indicate post-purchase regret or dissatisfaction (Gehring and Willoughby, 2002). Furthermore, pupillometry can monitor changes in pupil size in response to post-purchase experiences, reflecting emotional arousal and cognitive load (Bradley et al., 2008).

We suggest that actual consumer behavior across decision-making stages (Figure 2), encompassing both affective and behavioral components, can be evaluated using neuroscientific techniques that measure real-time neural responses. Tools such as EEG, fMRI, and eye-tracking provide insights into emotional (affective) reactions and cognitive processes during key stages like need recognition, evaluation, and post-purchase. For instance, EEG captures event-related potentials (ERPs) that reflect emotional and cognitive responses, while fMRI identifies brain regions involved in reward processing, attention, and decision-making. These methods offer precise, scientific insights into the interaction between emotions and behavior, providing a more accurate understanding of consumer actions than self-reported data or proxy measures.

## Findings

### Addressing neuromarketing's conceptual ambiguity

Given the definitional ambiguity surrounding neuromarketing and consumer neuroscience (Khamitov et al., 2020), our research adopts an interpretive approach to clarify what these terms encompass in practice and text, following Örtenblad's (2010) guidance. In line with Hulland and Houston (2020) and Palmatier et al. (2018), we aim to reduce ambiguity and define neuromarketing as “An interdisciplinary area which applies neuroscience and cognitive neuroscience to business. It is about creating brain-friendly content or communication which helps to understand how consumers react at non-conscious level in real time, based on brain operating principles and the responses can be measured by various neuro-metric or non-neuro metric techniques.” With advancing technology, marketers are increasingly able to influence and measure consumer perceptions and attitudes (Zhao, 2022). Our literature review shows that many foundational theories remain relevant and robust, even when applied to modern neuromarketing tools and techniques (Oliveira P. M. et al., 2022). Researchers have integrated key theories from consumer behavior, psychology, economics, and sociology with insights from consumer neuroscience to develop and test decision-making models. Table 2 presents several of these seminal theories used across contexts, supported by specific neuromarketing methods. Traditionally, marketing theories have emphasized behavioral intent as a predictor of action. However, this focus has faced criticism due to the intention-behavior gap—the disconnect between what consumers plan to do and their actual behavior (Sheeran, 2002). For example, a consumer may intend to purchase a product but change their mind at the point of sale due to price, availability, or competing options.

**Table 2:** Seminal theories and their mapping with purchase stages and attitudinal responses.

**References**	**Techniques**	**Theories**	**Pre-purchase**	**Purchase**	**Post -purchase**	**Affective (A)**	**Behavioral (B)**	**Cognitive (C)**
Alvino et al. (2020)	EEG + Questionnaire	NA		X		X	X	
Casado-Aranda et al. (2020)	fMRI + Questionnaire	Theory of consumer ethnocentrism	X			X		X
		Gaussian random field theory						
Cha et al. (2020)	fNIRS	Fast-diffusion model	X			X	X	X
		Sensory load theory						
		Perceptual load theory						
Fondevila i Gascón et al. (2020)	EDA + Questionnaire	Emotion contagion theory	X			X	X	
Hamelin et al. (2020)	ET + Facial + GSR + Questionnaire	ELM (elaboration likelihood model)	X			X		X
		HSM (heuristic systematic model)						
Hsu and Chen (2020)	EEG + Questionnaire	Dual process theory	X			X		X
Boscolo et al. (2021)	ET + Questionnaire	AIDA Model	X				X	X
Garczarek-Bak et al. (2021)	EEG + EDA + ET +Questionnaire		X				X	X
Gómez-Carmona et al. (2021)	fMRI	NA	X			X	X	X
Hakim et al. (2021)	EEG + ML + Questionnaire	ML Models	X				X	
Jai et al. (2021)	fMRI	SOR Theory	X			X	X	
Medina et al. (2021)	fMRI + Questionnaire	Attitude-behavioral gap	X			X	X	X
Mengual-Recuerda et al. (2021)	GSR + EEG	NA	X			X	X	
Rúa-Hidalgo et al. (2021)	FAC + GSR + ET	Emotional Assessment Theory	X			X	X	
Wajid et al. (2021)	EEG + moment-by-moment picture sort technique scale	SOR Model	X			X	X	X
		Dual system Model						
		Approach-avoidance Model						
Yun et al. (2021)	fMRI + Questionnaire	Evolution Theory	X			X	X	X
		Social Baseline Theory						
		Body-heart-mind Model						
		Free energy principle						
Zamith et al. (2025)	GSR + ET	NA	X			X	X	X
Zhang et al. (2021)	EEG (ERP) + IRT	Repetition Effect	X			X	X	X
		Repetition Suppression						
		Celebrity Effect						
Awan et al. (2022)	EEG + GSR + ECG	NA	X			X		X
Baldo et al. (2022)	HR + GSR + EEG + QUESTIONNAIRE	Davidson's influential approach-withdrawal motivation model		X		X	X	X
Casado-Aranda et al. (2022a,b)	fMRI + Questionnaire	Individual motivation theory	X			X	X	X
		Goal system theory						
Cirović et al. (2024)	EEG + ET + Questionnaire	Aristotelian persuasion theory	X			X	X	X
		Dual system approach theory of visual attention						
Gómez-Carmona et al. (2022)	fMRI + Questionnaire	Prospect theory	X					X
Gorin et al. (2022)	EEG + Questionnaire	Semantic theory		X		X		X
Hassani et al. (2022)	EEG	NA	X			X	X	X
Herrando et al. (2022)	GSR + EEG + Questionnaire	Emotion contagion	X			X		X
		Theory of arousal						
Herrando et al. (2022)	HRV + Survey	Flow theory/flow channel model	X			X		X
		SOR model						
Izadi et al. (2022)	EEG + Questionnaire + FGDs	SOR Theory		X			X	X
Kaklauskas et al. (2022)	TMS + fMRI + fEMG + ET	NA	X			X	X	X
Krampe (2022)	mfNIRS	NA	X				X	X
Li et al. (2022)	EEG		X			X		X
Pascucci et al. (2022)	ET + Questionnaire	Cue utilization theory	X			X		X
		Five- factor model						
Pozharliev et al. (2022a,b)	ET + EEG	Behavioral activation system (BAS)	X			X	X	X
		Behavioral inhibition system (BIS)						
		Dual coding theory						
		Social media influencer value model (SMIV)						
Savelli et al. (2022)	Implicit Priming Test +Eye-Tracking + EEG + Questionnaire + Focus Group	Signaling theory		X		X	X	X
Šola et al. (2022)	Eye- tracking + Facial Expression + IRT	Cognitive Processing				X	X	X
		Associative learning						
		Unconscious Processing						
Zhang and Lee (2022)	fMRI + IRT (behavioral test)	Consumer Attitude change theory	X			X	X	X
		Dual process theory						
Zhao (2022)	EEG + IRT + Questionnaire	SDL (Service Dominant Logic)	X					X
Juárez-Varón et al. (2023)	ET + Questionnaire	NA	X				X	
Kakaria et al. (2023b)	EEG + VR + Questionnaire	SOR Theory	X	X		X	X	X
Li et al. (2023b)	EEG	Excitation transfer theory	X			X		X
Oikonomou et al. (2023)	EEG	NA	X			X		X
Vela and Paredes (2023)	EEG + Questionnaire	NA	X			X	X	X
Xu et al. (2023)	EEG + GSR + Questionnaire	Brand Personality	X			X	X	X
Yüksel (2023)	ET	NA	X			X		X
Zahmati et al. (2023)	ET + Questionnaire	NA	X			X		X
Kaya et al. (2024)	EEG	NA	X			X		X
Lin et al. (2024)	EEG	NA	X			X	X	X
McInnes and Sung (2024)	Pupillometry	NA	X			X	X	X
Panda et al. (2024)	EEG	NA	X			X		X
Rancati et al. (2024)	IRT + Questionnaire	Customer Experience	X			X	X	X
Simonetti et al. (2024)	EEG + Questionnaire	NA	X			X		X
Šola et al. (2024)	ET	NA	X				X	X
Tan and Lee (2024)	fMRI + Questionnaire	NA	X				X	X
Ülker et al. (2025)	GSR + PPG + QUESTIONNAIRE	NA	X			X	X	X
Xu and Liu (2024)	EEG	NA	X	X		X		X
Zhang et al. (2024)	fMRI + Questionnaire	Consumer Wellbeing	X			X	X	X
Adalarasu et al. (2025)	EEG + Questionnaire	Emotional Intelligence	X				X	X
Khubchandani and Raman (2025)	ET + Questionnaire	SOR Theory	X			X		X
Lopez-Navarro et al. (2025)	EEG + EDA + Questionnaire	Perception	X			X		X
Marques et al. (2025)	EDA + FEA	ELM (Elaboration likelihood Model)	X			X	X	X
Šola et al. (2025)	ET	TAM	X				X	X
Yu et al. (2025)	fNIRS	ANTHROPOMORPHISM and PRO-SOCIAL BEHAVIOR	X				X	X

### Measured attributes

A comprehensive evaluation of the relative strengths and limitations of neuroscience research methods is often missing from the neuromarketing literature. Most studies tend to offer only brief descriptions of the specific neuroscience techniques they employ, without delving into their broader functional capabilities. As noted by Harris et al. (2018), there has been a lack of systematic analysis comparing neurometric and non-neurometric tools in terms of their effectiveness in measuring different psychological variables and their applicability across various domains. This review addresses that gap by providing a detailed overview of the full spectrum of neuroscience tools used in marketing research (see Table 3). It examines their respective advantages and disadvantages, and explores how these tools have been applied in consumer research to assess a range of constructs relevant to marketing and business—such as attention, emotion, liking, preference, and memory.

**Table 3:** Neuroscientific tools, their applications and measurement variables.

**Technology**	**Details**	**Insights**	**Application/Used**	**Measures**
**Neuro-metric Techniques**				
fMRI	fMRI measures haemodynamic activations i.e., changes in blood flow which is associated with changes in neural activity. Increased neural firing requires oxygen, which is delivered by blood, and the magnetic properties of oxygenated blood are different from those of non-oxygenated blood. This property is measured by fMRI as a distortion of the magnetic field generated by hydrogen protons.	fMRI being the most expensive and technical, requires maximum expertise and is generally carried out alone.	1. Sustainable Fashion Consumption 2. Branding 3. SNS Marketing 4. Healthcare decision making 5. AI acceptance 6. CTR behavior 7. CSR communication strategy 8. WOM effectiveness (after product harm crisis)	1. Memory and Desirability 2. Sensory Perception 3. Trust and Brand Engagement 4. Loyalty 5. Consumer Preference 6. Recall 7. Emotions Valence 8. Value Perception 9. Conflict resolution 10. Fear and reward processing 11. Risk perception 12. Prediction 13. Self-reflection 14. WTP 15. Decision-making 16. Aversive/negative affect processing
EEG	Electroencephalography records the electrical activity of the brain using electrodes placed on the scalp i.e., voltage fluctuations resulting from ionic currents within groups of neurons aligned in parallel on different frequencies. Measuring electrical activity from the brain is useful because it reflects how, many different neurons in the brain network communicate with each other via electrical impulses.	1. Researchers argue that EEG sensors are more effective as compared to galvanic skin response (GSR) and photoplethysmography (PPG) and fMRI as it allows us to get insights into both the moment-by-moment changes in emotional expression. 2. EEG can measure electrical signals in sub millisecond intervals, which has the highest temporal resolution among all the neuroimaging techniques. 3. Ambulatory EEG equipment can induce measure of error, thus, viable alternative to real-life experiences is to make use of the rapidly emerging VR technology, which allows for the creation of real-life-like experiences in a neuroscience laboratory setting. 4. The compatibility of VR technology with EEG recording systems allows for creating experiences with a higher degree of ecological validity and control.	1. Hospitality Marketing 2. PSS design and development 3. Food and Tourism Marketing 4. Neuro-architecture 5. Electric car preference 6. Rehabilitation Centers 7. Product Experience 8. SNS marketing 9. Branding 10. Media channel's effectiveness (print vs. TV) 11. Mobile advertising 12. Social advertising 13. Servicescape setting in retail 14. Influencer marketing 15. Sports marketing	1. Brand Choice or Selection 2. Cognitive and Affective State (Emotions) 3. Conflict 4. Cognitive Load 5. Engagement 6. Motivation 7. Frustration 8. Response inhibition 9. Creativity 10. Sleep patterns 11. Meditation or relaxation or reduced stress 12. Attention 13. Alertness 14. Preference Ranking 15. Attractiveness and Effectiveness of marketing communications 16. Emotion Valence 17. Perception 18. Memory 19. Face recognition 20. Prediction 21. Brand Association 22. Excitement 23. Interest 24. Approach - Avoidance Behavior
MEG	Magnetoencephalography (MEG) records the magnetic fields generated by neural activity. It detects and records the change of magnetic cerebral signals due to activities occurring on the cortical structures of the human brain when respondents are exposed to a stimulus with the assistance of a helmet placed on the respondents' scalp and with a great number of superconducting quantum interference detectors.	MEG has excellent time resolution and is often considered to capture deeper neural activity much better than EEG.	1. Food and tourism marketing 2. Advertising research	1. Perception 2. Attention 3. Memory 4. Preference 5. Emotional reaction 6. Tastes 7. Choice Behavior 8. Cognitive and affective appeal 9. Decision making
SST	SST is a variant of EEG, which measures the speed of information processing in the brain by detecting electrical potentials.	SST can be used to measure consumer's response to fast changing stimuli.	1. Advertisement	1. Sensory Perception 2. Valence of Emotion 3. Memory Effectiveness 4. Engagement 5. Face processing 6. Decision making 7. Attention
TMS	TMS is a brain stimulating method that aims to manipulate brain activities. It deactivates targeted brain regions through the pulsing of a strong electromagnet over that region, which induces an electric field in the underlying brain tissue and thus depolarizes neurons in order to alter brain activity. TMS can both facilitate and disrupt brain activity of particular sets of neurons to test their causality.	TMS-EEG can be used as an “excitability probe,” with the EEG output reflecting the excitability of the underlying cortex at the time the TMS pulse was delivered.	1. Approach – avoidance conflict 2. Cognitive and affective decision making 3. Consumer preference	1. Task Performance 2. Causal role of a cortical region 3. Facial Identity 4. Decision Making
fNIRS	“Functional near-infrared spectroscopy (fNIRS) – fNIRS measures variations in NIR absorption to observe haemodynamic variations, i.e., changes in the concentrations of oxy-Hb, deoxy-Hb, and total Hb in certain functional cortices. NIR ranges in wavelength from 650 to 950 I, and is primarily absorbed by oxy-Hb and deoxy-Hb, rather than by other biological tissues.	Existing fNIRS studies have identified activation patterns in the prefrontal cortex during product selection (Kumagai, 2012), risk assessment, financial investment (Shimokawa et al., 2009), price evaluation and price prediction (Mitsuda et al., 2012) tasks (Cakir et al., 2018). fNIRS is more suitable for studies of subjects in daily-life contexts and 2 fNIRS devices can be linked, shared and synchronized.	1. Product Selection 2. Risk Assessment 3. Financial investment 4. Price Evaluation 5. Price prediction 6. Advertising preference	1. Decision making Process 2. Attention 3. Cortical Activations 4. Preferences
PET	PET is a nuclear imaging technique based on gamma radiation caused by decaying radionuclides which are inserted into the body of the respondent. It measures modified molecules in body tissues.	It is unlikely to be used in future researches of consumer neuroscience and neuromarketing due to use of radioactive substance, which needs to be taken by the participants.		1. Memory encoding 2. Emotional Engagement 3. Engagement 4. Attention 5. Processing Visual Input 6. Recall
**Non-Neuro metric Techniques**				
Eye-Gaze Tracking	Eye tracking is the recording of eye position (gaze point) and movement on a 2D screen or in 3D environments based on the optical tracking of corneal reflections. Near-infrared light is directed toward the center of the eyes (the pupils) causing visible reflections in the cornea (the outer-most optical element of the eye), which are tracked.	Eye tracking often complemented by other biometric sensors to understand behavior based on when (and which) cognitive and emotional responses are activated. Also, the amount of dilation is proportional to the strength of arousal, so if we are tracking pupil dilation, it can be used to capture emotion arousal.	1. SNS marketing 2. Product development and performance 3. Product and package design 4. Customer Experience 5. In-store navigation 6. Self-design/self lay-out 7. Driving simulators 8. Dashboard design 9. Website usability, design, and testing 10. Mobile Apps usability, design, and testing 1 1. Advertisement testing/effectiveness 12. Classroom Attention and distractions 13. Gaming and UX testing 14. Food and Tourism Marketing 15. Weak and Strong Brands 16. Influencer Marketing (increase followers) 17. Sporting and sponsorship events 18. Mobile Advertising 19. Environmental attitude change and retention 20. Social Communication	1. Visual Attention 2. Fixation and Gaze Positions 3. Eye Movement Pattern 4.Spatial Resolution 5. Excitement 6. Pupil Dilation 7. Interest and Preferences
GSR/EDA/SCR	GSR originates from the autonomic activation of sweat glands in the skin. The sweating on hands and feet is triggered by emotional stimulation. It reflects the variation in the electrical characteristics of the skin.	While GSR is an ideal measure to track emotional arousal, it is not able to reveal the emotional valence, that is, the quality of the emotions, thus GSR needs to be complemented by other biometric sensors to capture the full picture of human behavior.	1. Content, product, or service testing 2. Sensory arousal 3. Personality characteristics 4. Consumer Preference 5. Media, Movies and Ad testing 6. Usability of Apps, Websites 7. Sports 8. FBO's strategic decision making 9. Mobile Advertising 10. Environmental Attitude change and retention 11. Online Customer review 1 2. Social Communications	1. Emotional Engagement/state 2. Arousal of Emotions 3. Attention 4. Habituation 5. Anticipation 6. Cognitive effort 7. Stress 8. Fear 9. Anxiety 10. Purchase Intentions
Facial Action Coding	It is a fully standardized classification system of facial expressions for expert human coders based on anatomic features or by a facial expression software.	While facial recognition delivers valuable information on the quality of an emotional response, generally referred to as its valence, one core limitation of computer-based facial coding lies in its inability to assess someone's emotional arousal, that is, the intensity of an emotion, thus it needs to be complemented by other biometric sensors to capture the full picture of human behavior.	1. Customer Preference 2. Persuasive communication 3. Customer STP 4. Media testing and advertisements 5. Software UI and website design and testing 6. Avatar marketing 7. SNS Marketing 8. Driving attitude and behavior 9. Mobile advertising 10. Environmental Attitude change and retention 11. Social Communications	1. Unconscious reactions 2. Emotions Valence 3. Attention based on head position 4. Engagement 5. Attitude and behavior 6. Approach-withdrawal response
FACIAL EMG (FEMG)	To track the activity of facial muscles with electrodes attached to the skin surface. fEMG detects and amplifies the tiny electrical impulses generated by the respective muscle fibers during contraction. The most common fEMG sites are in proximity to the following major muscle groups – Involuntary muscle group – Right/left corrugator rinkleia (“eyebrow rinkle”) and orbicularis occuli (lower eyelid) Right/left zygomaticus (major)			
HR (Heart Rate) or HR Variability (HRV)	Heart rate or number of times the heart beats has been used as an indicator of attention and arousal. Heartbeats generate electrical activity, which can be captured by placing two electrodes on the wrists. The time between two consecutive heartbeats is called the inter-beat interval and HRV corresponds to the rate of firing of the vagus nerve, relates to individuals' ability to return the body to a low-arousal state after an emotional stimulus.	Heart rate acceleration indicates increased arousal (Wang et al., 2015) while heart rate deceleration indicates increased attention and interest. HRV IS more suitable for video stimuli but not still images. HRV was assessed as a proxy measure of vigilance or cognitive effort.	• Hospitality, Tourism and Leisure Marketing • FBO's strategic decision making • Mobile advertising • Flow in Social Commerce • Social Communications	1. Emotional Engagement/state of mind 2. Arousal of Emotions 3. Inter-beat HR–> emotional valence 4. Attention 5. Stress Levels 6. Anxiety and Fear 7. Cognitive load 8. Affective states
IRT/IPT/IAT	It measures conscious and unconscious response times to external stimuli and relies on latency measures. Conscious responses occur 500 ms after a stimulus is presented, and unconscious reactions are below the 500-ms threshold, which renders verbalisation impossible. The stronger two concepts are associated in the mind, the faster and more accurate will be the response.		1. Food and Tourism Marketing 2. Packaging 3. Branding Brand attitudes 4. Tobacco Consumption 5. SNS Marketing	1. Reaction Time 2. Underlying Evaluations/Attitudes 3. Preference 4. Decision Making 5. WTP 6. Memory

Consumer research represents one of the most prominent areas where neuroscientific methods have been applied extensively (Lim, 2018), underscoring their potential to complement—or even surpass—traditional, consciousness-based research tools. Neuromarketing techniques have been used to measure a range of variables, including affective responses, emotional valence, arousal, cognitive load, and other cognitive processes. Table 4 presents key metrics that neuroscientific methods can reliably capture—many of which are difficult to assess using self-reported data alone. By integrating multiple technologies, neuromarketing enables a comprehensive understanding of consumer decision-making, encompassing cognitive, emotional, and behavioral aspects. For instance, electroencephalography (EEG) and functional magnetic resonance imaging (fMRI) reveal neural activity associated with attention, memory, and emotion, while eye-tracking identifies visual attention and engagement. Likewise, galvanic skin response (GSR) and heart rate variability (HRV) provide indicators of physiological arousal and emotional intensity. When combined, these tools offer rich, multidimensional insights into how consumers perceive, evaluate, and respond to marketing stimuli. For example, the integration of eye-tracking with EEG can link visual attention to brain activity, helping to identify which elements of a product or advertisement most strongly influence purchase intent. Such multi-modal approaches bridge the gap between conscious and unconscious behavior, enabling researchers to trace the full consumer journey—from initial exposure to post-purchase reflection—while offering actionable insights for marketers.

**Table 4:** Measurement indicators.

**Measures**	**Pre-purchase**						**Purchase**						**Post purchase**					
	**fMRI**	**EEG**	**ET**	**GSR**	**FE**	**IR**	**fMRI**	**EEG**	**ET**	**GSR**	**FE**	**IR**	**fMRI**	**EEG**	**ET**	**GSR**	**FE**	**IR**
Attention	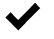	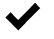	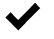	X	X	X	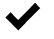	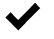	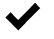	X	X	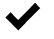	X	X	X	X	X	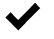
Engagement	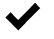	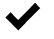	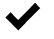	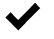	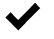	X	X	X	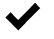	X	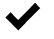	X	X	X	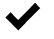	X	X	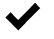
Cognitive load	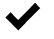	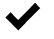	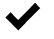	X	X	X	X	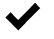	X	X	X	X	X	X	X	X	X	X
Emotion valence	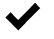	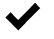	X	X	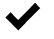	X	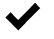	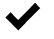	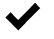	X	X	X	X	X	X	X	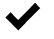	X
Emotion arousal	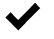	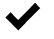	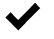	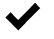	X	X	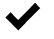	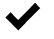	X	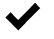	X	X	X	X	X	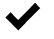	X	X
Preference	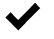	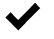	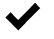	X	X	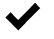	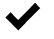	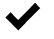	X	X	X	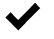	X	X	X	X	X	X
Memory	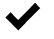	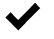	X	X	X	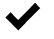	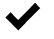	X	X	X	X	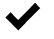	X	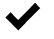	X	X	X	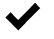

### Measurement tools and techniques

Marketing focuses on influencing consumer perceptions and decisions, while neuroscientific tools enable us to gain insights into the brain's responses to marketing stimuli. Three methods are typically employed to gauge customers' reactions: behavioral measures, self-reports, and psychophysiological tests (Hsu and Chen, 2020). Due to limitations and criticism of traditional market research methods like self-report, focus-group etc., has made these methods insufficient to truly capture the true responses (Boz et al., 2017; Nemorin, 2017; Bastiaansen et al., 2019; Boscolo et al., 2021; Casado-Aranda et al., 2020; Yu et al., 2023; Zhang and Lee, 2022). These approaches are criticized for biases such social desirability, lack of articulation, remembering correctly and exactly, misinterpretation, manipulation, and intuitive ‘knowing' (Dowling et al., 2020). Gorin et al. (2022), Hsu and Cheng (2018), and Yun et al. (2021) contend that standard techniques cannot capture emotional preferences or implicit/internal psychological mechanisms at the unconscious level or reflect genuine behavior. They mostly use conscious answers, which may be cognitively biased or socially influenced (Wajid et al., 2021). Most traditional methods use *post-hoc* evaluations of psychological reactions to marketing stimuli, which vary by time, effort, and environment. These methods can tell if a person is positive, negative, aroused, or willing to approach or avoid something (Verhulst et al., 2019).

From the consumer's standpoint, marketing and consumer research have increasingly employed both neurometric (e.g., fMRI, EEG, MEG, SST, TMS, fNIRS, and PET) and non-neurometric (e.g., Eye Tracking, Galvanic Skin Response, Facial Action Coding, facial EMG, Heart Rate, and Infrared Thermography) techniques to better understand behavior—particularly decision-making processes. These tools enable researchers to uncover cognitive, emotional, and behavioral responses, helping to interpret, explain, and address barriers to acceptance and action regarding various issues. The emergence of interdisciplinary fields such as neurophilosophy, neuroeconomics (Sanfey et al., 2006), neurofinance, and neuromarketing reflects the need to move beyond traditional research approaches, which often answer what is happening but fall short in explaining why or how it occurs (Medina et al., 2021). Most literature reviews in this area incorporate primary data from neurometric, non-neurometric, and self-reporting techniques, with many emphasizing the value of triangulating these methods alongside conventional research strategies. This integration—combining neuroscience-based tools with traditional methodologies—enhances the reliability, validity, and generalizability of consumer insights (Boz et al., 2017; Zhang and Lee, 2022). As reflected in Table 5, most studies adopt a hybrid approach, combining advanced techniques with standard research practices to achieve a more holistic understanding of consumer behavior.

**Table 5:** Instruments and techniques utilized in reviewed studies.

**References**	**Neuro-metric**			**Non-Neuro metric**					**Traditional techniques**
	**fMRI**	**EEG**	**fNIRS**	**ET**	**GSR**	**Facial**	**Implicit tests**	**HR**	
Alvino et al. (2020)		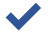							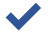
Casado-Aranda et al. (2020)	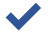								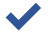
Cha et al. (2020)			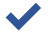						
Fondevila i Gascón et al. (2020)					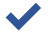				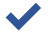
Hamelin et al. (2020)				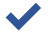	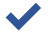	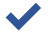			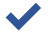
Hsu and Chen (2020)		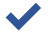							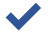
Boscolo et al. (2021)				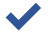					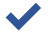
Garczarek-Bak et al. (2021)		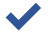		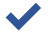	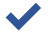				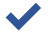
Gómez-Carmona et al. (2021)	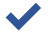								
Hakim et al. (2021)		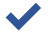							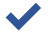
Jai et al. (2021)	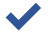								
Medina et al. (2021)	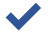								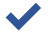
Mengual-Recuerda et al. (2021)		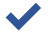			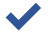				
Rúa-Hidalgo et al. (2021)				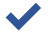	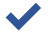	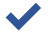			
Pelowski et al. (2020)			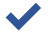						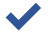
Wajid et al. (2021).		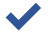							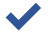
Yun et al. (2021)	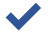								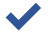
Zamith et al. (2025)				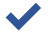	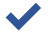				
Zhang et al. (2021)		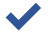					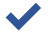		
Awan et al. (2022)		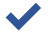			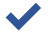			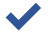	
Baldo et al. (2022)		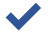			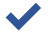			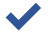	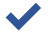
Casado-Aranda et al. (2022a,b)	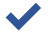								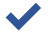
Cirović et al. (2024)		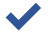		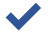					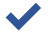
Gómez-Carmona et al. (2022)	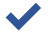								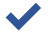
Gorin et al. (2022)		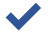							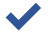
Hassani et al. (2022)		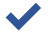							
Herrando et al. (2022)		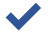			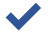				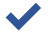
Herrando et al. (2022)								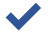	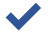
Izadi et al. (2022)		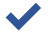							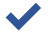
Kaklauskas et al. (2022)	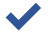			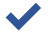		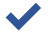			
Krampe (2022)			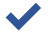						
Pozharliev et al. (2022a)		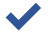		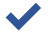					
Pozharliev et al. (2022b)		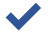							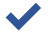
Savelli et al. (2022)		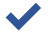		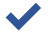			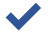		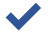
Šola et al. (2022)				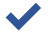		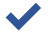	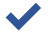		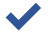
Zhao (2022)		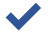					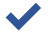		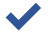
Zhang and Lee (2022)	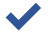						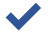		
Juárez-Varón et al. (2023)				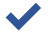					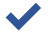
Kakaria et al. (2023b)		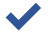							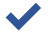
Li et al. (2023b)		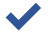							
Oikonomou et al. (2023)		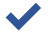							
Vela and Paredes (2023)		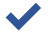							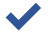
Xu et al. (2023)		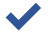			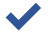				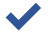
Yüksel (2023)				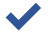					
Zahmati et al. (2023)				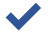					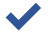
Kaya et al. (2024)		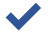							
Lin et al. (2024)		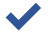							
Lyulyov et al. (2024)		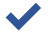							
Panda et al. (2024)		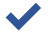							
Rancati et al. (2024)							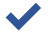		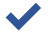
Simonetti et al. (2024)		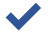							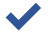
Šola et al. (2024)				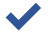					
Tan and Lee (2024)	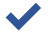								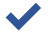
Ülker et al. (2025)					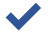				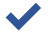
Xu and Liu (2024)		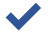							
Zhang et al. (2024)	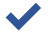								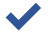
Adalarasu et al. (2025)		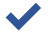							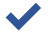
Khubchandani and Raman (2025)				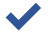					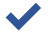
Lopez-Navarro et al. (2025)		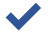			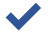				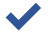
Marques et al. (2025)					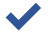	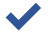			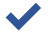
Šola et al. (2025)				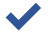					
Yu et al. (2025)			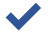						

### NeuroTypology 3 × 3

**Figure 3:**
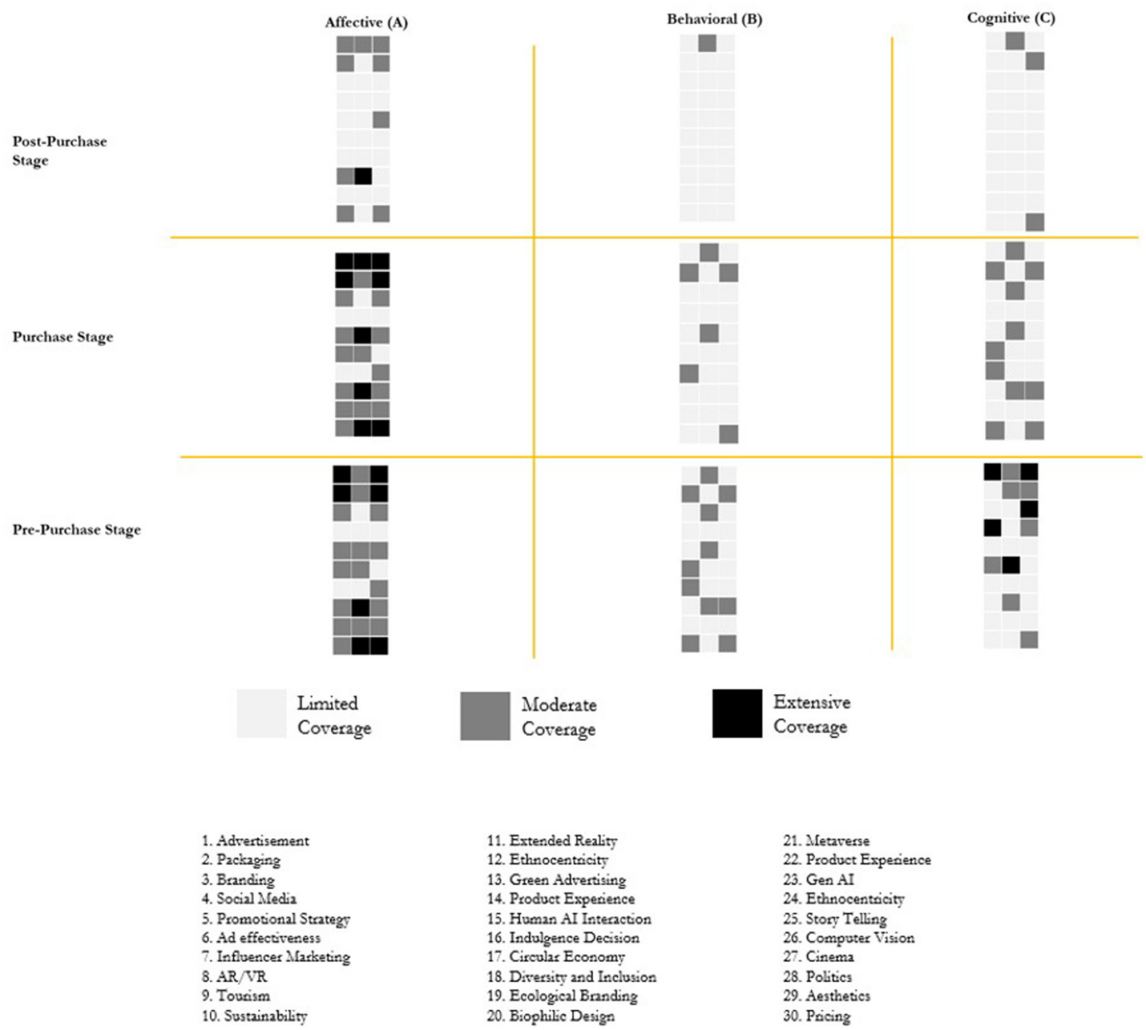
Proposed 3 × 3 typology.

The study introduces a 3 × 3 typology (Figure 3) that focuses on the various stages of consumer purchase decisions. This framework is structured along two dimensions: (1) decision-making stages—Pre-purchase, Purchase, and Post-purchase—and (2) components of attitude—Affective, Behavioral and Cognitive. The typology integrates insights from both neuromarketing and consumer decision-making literature, employing these as “method theories” (Jaakkola, 2020) to offer new perspectives on each stage of the purchase process. The Y-axis represents the stages of decision-making, while the X-axis denotes the level of level of affective, behavioral and cognitive engagement. Each quadrant in the typology includes a schematic representation of literature coverage related to specific functional domains. In total, 30 functional domains are outlined, with each corresponding to an individual block—for instance, Advertising is represented as the first block, Packaging as the second, and Pricing as the thirtieth. The intensity of research in each domain is visually indicated through block shading: light gray signifies fewer than 20 research papers, dark gray represents 20–40 papers, and black indicates more than 40 papers.

**Figure 4:**
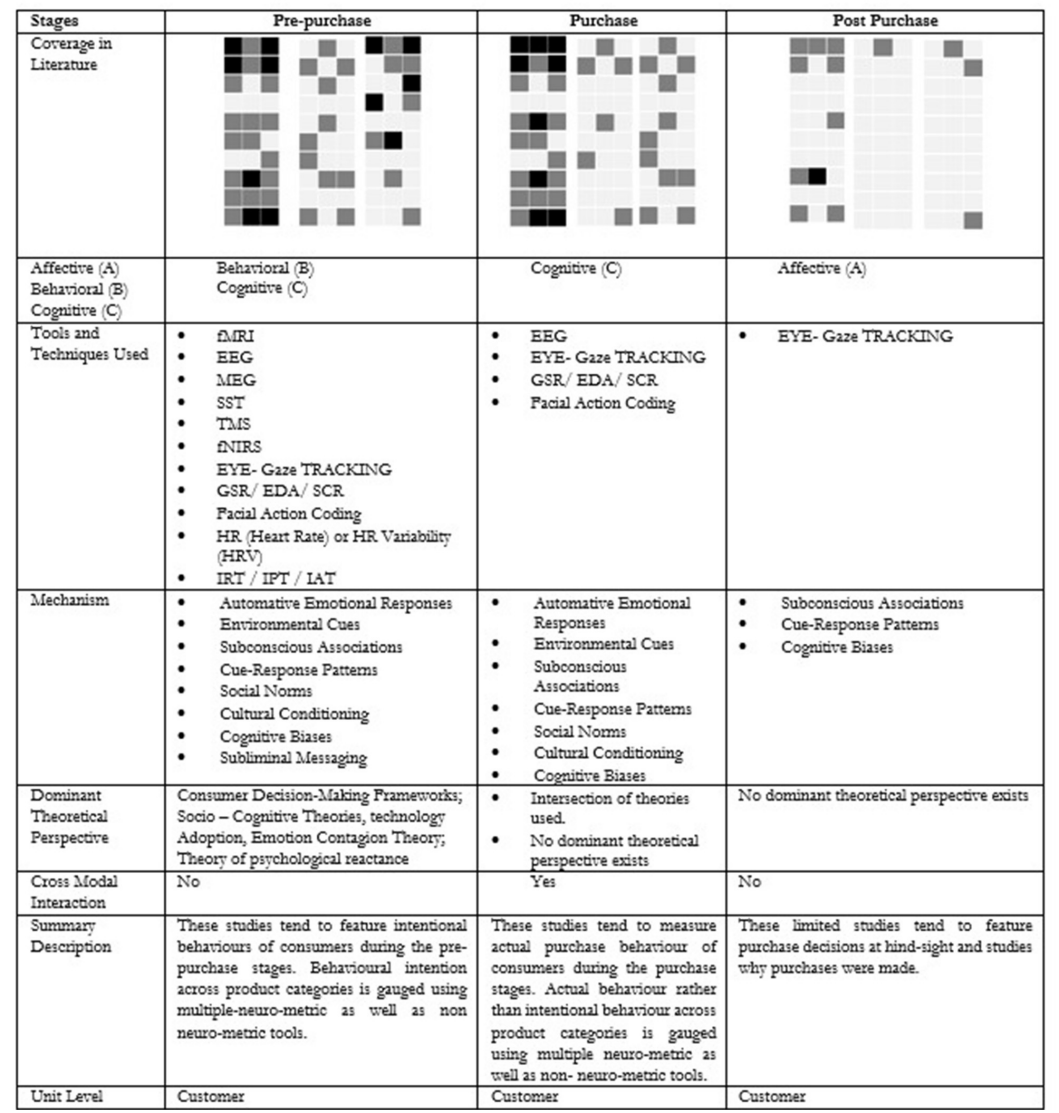
Literature coverage using 3 × 3 typology.

Given the critical role of unconscious decision-making in consumer behavior—an area that has gained growing recognition in marketing literature—contemporary research increasingly suggests that a substantial portion of purchasing behavior is influenced by unconscious processes. The proposed typology (Figure 4) illustrates various mechanisms through which these unconscious processes via the affective, behavioral and cognitive components can shape purchase decisions. For instance, automatic emotional responses are swift, involuntary reactions to stimuli that occur without conscious deliberation. Such responses may be triggered by visual elements, sounds, scents, or past experiences associated with a brand or product. Often, these emotional reactions precede rational analysis, leading consumers to make decisions based on immediate feelings rather than deliberate thought. This phenomenon is supported by Damasio's (1994) somatic marker hypothesis, which posits that emotions are deeply intertwined with decision-making. According to this theory, emotional responses to specific stimuli create “somatic markers”—neural patterns stored in the brain that guide future behavior in similar contexts. These markers are automatically activated, subtly steering choices and actions. Additional support comes from Winkielman et al. (2005), who showed that even subliminal exposure to emotional stimuli can influence consumer preferences and decision-making. In a similar vein, the proposed typology emphasizes how prior research has employed various mechanisms to assess unconscious decision-making across different stages of the consumer journey. It also identifies specific neurometric tools aligned with each decision-making stage. Overall, the affective, behavioral, and cognitive components of unconscious decision-making play a crucial role in influencing consumer behavior. By acknowledging and harnessing these underlying processes, marketers can craft strategies that connect with consumers on a deeper, more instinctive level, ultimately enhancing the effectiveness of their influence.

## Future research areas and applications

Applications of consumer neuroscience can be broadly categorized into two types: fundamental and applied. Fundamental applications aim to advance theoretical understanding by developing and refining models based on specific phenomena and variables within controlled environments. These studies explore the relationships between dependent and independent variables, often with potential commercial implications. In contrast, applied applications address real-world business challenges, involving real-time data collection and experimentation to inform managerial decision-making. While fundamental research emphasizes theoretical contributions, applied research is focused on generating actionable insights for business practice. Table 6 outlines the major domains of consumer neuroscience, detailing both fundamental and applied uses. It also illustrates how various neurometric and non-neurometric tools are employed to measure constructs within these applications.

**Table 6:** Use cases in neuromarketing.

	**Generic application**	**Tools used**	**References**
Advertisement	Ad preference	EDA + FEA	Marques et al., 2025
	Advertising previews on Online Social Network (OSN)	Eye- tracking + Facial Expression + Implicit response	Šola et al., 2022
	AD Effectiveness	HR + GSR + EEG	Baldo et al., 2022
	Online advertisement	fMRI	Casado-Aranda et al. (2022a,b)
	Gender based preferences for Advertisement	ET	Boscolo et al., 2021
	Advertisement effectiveness	EEG	Hassani et al., 2022
	AD Effectiveness	GSR + ET	Zamith et al., 2025
	Destination Advertisement	EEG	Li et al., 2023b
	Ad effectiveness; endorsement	ET	Zahmati et al., 2023
	AD Effectiveness for emotional resonance	fNIRS	He et al., 2021
	Promotional strategies for Sports marketing	EEG	Izadi et al., 2022
	Advertising effectiveness	EEG	Simonetti et al., 2024
	Fund-raising; donation	fNIRS	Yu et al., 2025
	AD endorsement	EEG	Adalarasu et al., 2025
Environment/ sustainability	Ecological branding strategy	Review	González-Morales et al., 2020
	Environmentally sustainable marketing communication	EEG + ET	Cirović et al., 2024
	Green advertising message	fMRI	Gómez-Carmona et al., 2022
	CSR Communication Strategy/Consumption of SRPs	fMRI	Medina et al., 2021
	Story-telling/Pro-environment attitude change	ET + Facial + GSR	Hamelin et al., 2020
Branding	Brand Choice Prediction	EEG + EDA + ET	Garczarek-Bak et al., 2021
	Ecological Branding Strategy	Review	González-Morales et al., 2020
	Brand Personality	EEG + GSR	Xu et al., 2023
	Brand Association	EEG	Gorin et al., 2022
Product	PSS design and development for Value Cocreation and Conflict Resolution	EEG + Implicit Response	Zhao, 2022
	Product Presentation	fMRI	Jai et al., 2021
	Enthnocentric Consumer Behavior via product labels	fMRI	Casado-Aranda et al., 2020
Tourism and Hospitality	Hospitality/Gastronomy	EEG + GSR	Mengual-Recuerda et al., 2021
	Communication strategy for Food and Tourism Industry	Implicit Priming Test +Eye-Tracking + EEG + Questionnaire + Focus Group	Savelli et al., 2022
	Tourism	EEG	Vela and Paredes, 2023; Li et al., 2023a
	Hotel Selection/Hospitality Marketing	EEG	Hsu and Chen, 2020
Consumer research	Customer experience	IRT	Rancati et al., 2024
	Preference prediction of food	EEG+ ML	Hakim et al., 2021
	Food ordering	ET	Khubchandani and Raman, 2025
	Product Experience via Wine tasting	EEG	Alvino et al., 2020
	Customer sensory experience	fNIRS	Cha et al., 2020
	Food Pleasantness	fMRI	**Gómez-Carmona et al.**, **2021**
	Human-AI Interaction or medical decision making	fMRI	Yun et al., 2021
	Decision making	TMS + fMRI + fEMG + ET	Kaklauskas et al., 2022
	Medical – AI	fMRI	Zhang et al., 2024
	Virtual reality	EEG + VR + Questionnaire	Kakaria et al., 2023b
	Fashion	ET	Juárez-Varón et al., 2023
	Customer privacy	fMRI	Tan and Lee, 2024
	Political Campaign	ET	Šola et al., 2025
	Virtual Reality; Retail Shopping	GSR + PPG	Ülker et al., 2025
	Customer Choice	EEG	Panda et al., 2024
Digital Marketing	SNS (Social Networking Services) marketing	EEG + IRT	Zhang et al., 2021
	SNS (Social Networking Services) marketing - Click Through marketing	fMRI + IRT	Zhang and Lee, 2022
	Social Media Marketing	EEG	Wajid et al., 2021
	Social Media Marketing	FAC + GSR + ET	Rúa-Hidalgo et al., 2021
	Online Customer reviews	GSR + EEG	Herrando et al., 2022
	Social Commerce	HRV	Herrando et al., 2022
	Influencer Marketing	EDA + ET + EEG	Fondevila i Gascón et al., 2020; Pozharliev et al., 2022a,b
	E-Commerce	ET	Yüksel, 2023
	Online customer reviews	ET	Šola et al., 2024

As neuromarketing continues to advance, there are several promising avenues for future research to deepen our understanding of consumer behavior. First, integrating multiple neuroimaging techniques—such as fMRI, EEG, and eye-tracking—can offer a more holistic view of the decision-making process. By combining these methods, researchers can simultaneously capture neural, physiological, and attentional data, providing richer insights into how consumers respond to various stimuli in different contexts. Second, the use of advanced machine learning algorithms and artificial intelligence can help decode complex patterns underlying consumer behavior. By applying deep learning models to multimodal data—including brain signals, eye-tracking, facial expressions, and other physiological indicators—researchers can more accurately predict purchasing decisions, preferences, and emotional reactions (Venkatraman et al., 2015). Multimodal data offers a more comprehensive representation of human cognition and emotion compared to isolated data sources (Usman et al., 2025). Deep learning models are particularly well-suited for uncovering complex, non-linear relationships across varied inputs—such as neural signals, gaze patterns, and facial expressions—enabling more precise predictions of consumer preferences, decisions, and emotional responses (Marques dos Santos and Marques dos Santos, 2024). This integrated approach mirrors the brain's natural ability to process information through multiple channels, leading to richer and more comprehensive insights into human behavior. It also supports the creation of more personalized and impactful marketing strategies. Third, future research should explore the unconscious processes that influence consumer behavior. While much of the current literature focuses on conscious decision-making, many purchasing choices are shaped by subconscious factors. Incorporating tools like implicit association tests (IATs) alongside neural measurements could reveal these hidden drivers of behavior. Fourth, extending neuromarketing research to diverse and naturalistic settings is crucial. Most current studies are conducted in controlled laboratory environments, which may not fully capture real-world consumer behavior. While lab studies allow for precision and control, they often lack ecological validity, limiting the generalizability of findings to real-life contexts. Portable and wearable neuroimaging devices, such as mobile EEG, mobile eye-tracking glasses, HRV monitors, and EDA sensors, can be employed to study consumer responses in dynamic environments like retail stores, shopping malls, or even during outdoor advertising exposure. These tools enable researchers to collect real-time, context-rich data, capturing spontaneous consumer reactions as they naturally occur. Integrating such technologies allows for a more comprehensive understanding of how environmental factors, social influences, and emotional triggers shape consumer decision-making in everyday life. This approach not only enhances the relevance of neuromarketing insights for practitioners but also bridges the gap between academic research and practical application in real-world marketing strategies.

**Figure 5:**
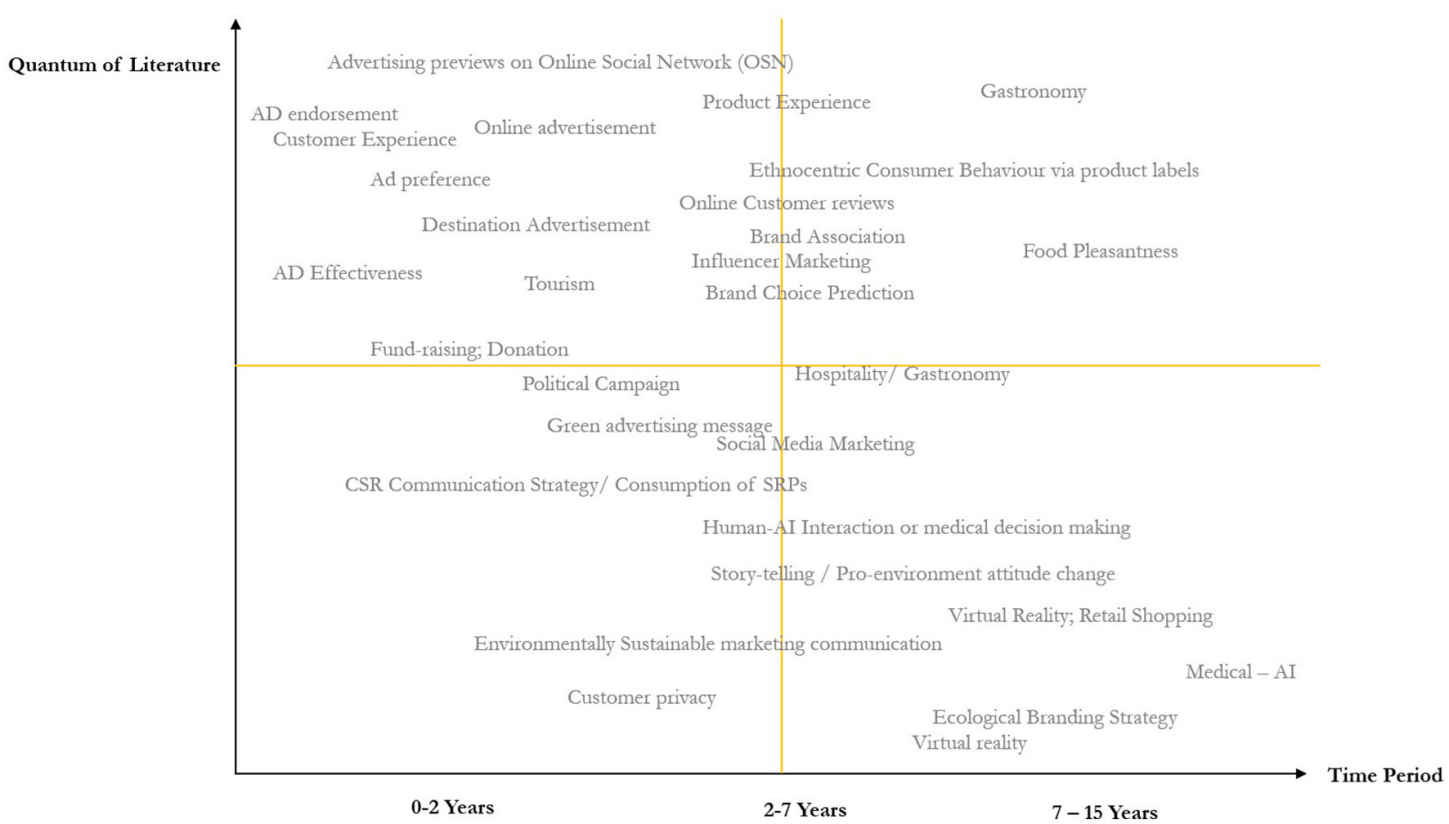
Potential future areas.

There are several underexplored areas that could emerge as important research topics in neuromarketing (Figure 5). Neuroscience offers valuable tools for studying attention, attitudes, emotions, and memory-based decision-making, all of which can be leveraged in neuromarketing. In particular, neuromarketing has the potential to address social marketing challenges, such as excessive drinking, drug use, and climate change. Public safety campaigns, which are essentially marketing initiatives, could greatly benefit from these methods (Stanton et al., 2017). We argue that neuromarketing can have positive implications for both society and consumers—an aspect often overlooked in ethical discussions surrounding the field (Stanton et al., 2017). Additionally, there is a need for more international and cross-cultural research to understand how consumers from diverse countries and cultures engage with neuromarketing technologies. Future studies could validate and expand the generalizability of neuromarketing findings by exploring how regulations and cultural contexts differ across nations and societies.

## Discussion

As consumer behavior rapidly evolves and technology advances, the digital landscape becomes increasingly complex, presenting a significant challenge for marketers to understand the emotional and cognitive drivers behind consumer decisions. Traditional marketing methods, such as surveys and focus groups, often fail to capture the subconscious factors that influence purchasing choices. In today's fast-paced market, where attention spans are shorter and consumer options are plentiful, neuromarketing provides a valuable approach for creating more targeted, personalized, and effective marketing strategies. A systematic review of the neuromarketing literature reveals a multifaceted landscape. Previous research has employed various methodologies to explore the role of neuromarketing in marketing studies. These studies have also highlighted gaps in the literature, such as the need to clarify the definition of neuromarketing, investigate emerging subfields within the discipline, and assess the effectiveness of various neuromarketing tools (Lin et al., 2018; Oliveira Í. A. et al., 2022). To address these gaps, we conducted a systematic literature review, aiming to deepen the understanding of neuromarketing as an evolving and contemporary field. This review aims to summarize the integration of neuroscience into consumer behavior research, commonly referred to as “neuromarketing,” which provides valuable insights into the neural mechanisms driving consumer actions. It highlights the various stages of consumer purchasing and examines brain responses at each stage. Unlike previous reviews, our framework incorporates findings from all three stages—pre-purchase, purchase, and post-purchase. Techniques such as eye-tracking, fMRI, and EEG have been shown to reveal neural triggers that influence preferences and intentions (Lee et al., 2007). Our systematic review and synthesis focus on empirical research, commercial applications, and theoretical development, with the goal of establishing a standardized definition for neuromarketing and addressing the definitional ambiguity that exists in the field. Additionally, we propose an integrated conceptual framework that maps neurometric tools to the consumer purchase stages. This study introduces a 2 × 3 typology, which combines decision-making processes (conscious vs. unconscious) with the buying stages (pre-purchase, purchase, and post-purchase). This framework offers a roadmap for scholars to explore under-researched areas in neuromarketing and identify gaps in the existing literature. The typology also facilitates comparisons of different neuromarketing techniques, tools, or theories, promoting more rigorous and systematic research. To the best of the author's knowledge, this is the first review to examine actual behavior (rather than proxy or intentional behavior) across decision-making stages, using both neurometric and non-neurometric tools. Moreover, the study emphasizes several neurometric tools that have effectively measured consumer behavior at these stages, offering an alternative to relying on proxy variables. This review makes significant contributions to the field by synthesizing a broad range of knowledge and providing a comprehensive understanding of neuromarketing. It supports the study of consumer behavior at different stages of the buying process by identifying key themes, tools, techniques, and measurement variables that influence purchasing decisions. Furthermore, it highlights the growing role of unconscious decision-making, deepening our understanding of how both conscious and unconscious behaviors shape decision-making. The review also identifies promising research directions and introduces a 2 × 3 framework to measure actual consumer behavior across various decision-making stages, along with the most effective tools for capturing these behaviors. Finally, the review points out existing gaps in the literature and offers valuable insights for advancing future research in neuromarketing.

## Implications of the study

The findings of this review offer both theoretical and practical implications. First, our systematic analysis and synthesis of the literature contribute to empirical research, commercial applications, and theory development, helping to establish a standardized definition for neuromarketing and addressing its definitional ambiguity. Second, we explore how consumer neuroscience integrates existing theories and frameworks to better understand both intentional and actual consumer behaviors. This study provides a thorough review of how actual behavior is assessed and measured across the five stages of consumer decision-making: (1) need recognition, (2) information search, (3) evaluation of alternatives, (4) choice, and (5) post-purchase. Third, the study introduces a 2 × 3 typology that combines decision-making (conscious vs. unconscious) with the buying stages (pre-purchase, purchase, and post-purchase). This typology serves as a framework for researchers to explore underexplored areas in neuromarketing and identify gaps in the existing literature (Oliveira Í. A. et al., 2022; Zhang and Lee, 2022; Li et al., 2022; Levallois et al., 2021; He et al., 2021). Additionally, it provides a means to compare and combine different neuromarketing techniques, tools, or theories, promoting more rigorous and systematic research. Fourth, to the best of the authors' knowledge, this is the first review to examine actual behavior across decision-making stages using both neuro-metric and non-neuro-metric tools. The study highlights various neurometric tools and techniques that have been effectively used to measure actual consumer behavior at different buying stages, offering an alternative to proxy variables. The review also offers several practical implications. It provides valuable insights for product managers, brand managers, retailers, and advertisers by identifying existing and emerging tools mapped to different stages of the consumer decision-making process. Specifically, it emphasizes how brain activity and emotional responses are linked to various stimuli, helping marketers gain a more accurate understanding of consumer feelings toward products, instead of relying on potentially biased self-reported data. By examining the functional capabilities of the tools discussed, marketers can optimize their campaigns, enhance product design, and create more effective brand strategies. Ultimately, this review supports marketers in transitioning toward data-driven strategies informed by neuroscience.

## Limitations

While this study addresses several important aspects of the neuromarketing literature, it does have certain limitations. First, many neuromarketing studies tend to oversimplify consumer behavior by focusing mainly on neural or emotional responses, often neglecting the complex nature of decision-making, including cultural, social, and cognitive factors. Future research exploring the impact of socio-cultural influences would enrich these findings. Second, expanding neuromarketing research to more diverse and naturalistic settings is crucial. Most current studies are conducted in controlled laboratory environments, which may not fully capture real-world consumer behavior. The use of portable neuroimaging tools, such as mobile EEG, could allow researchers to study consumer responses in more dynamic, real-world contexts like retail stores, leading to more ecologically valid data. Additionally, this review used a domain-based systematic literature review approach, which primarily focuses on publication volume and theoretical perspectives. Future research could benefit from employing alternative review methods, such as theory-based reviews, method-based reviews, bibliometric analysis, or content analysis, to gain more in-depth insights into the topic. This review also included only Q1-ranked journals to ensure a consistent level of academic rigor and theoretical contribution. While this enhances the reliability of insights drawn, it may exclude emerging or interdisciplinary work published in lower-ranked journals. Future reviews could expand this scope to include Q2 and field-specific journals to capture the full breadth of evolving contributions in neuromarketing.

## Data Availability

The original contributions presented in the study are included in the article/Supplementary material, further inquiries can be directed to the corresponding author.
