# Supplementary material for: Neuro-insights: a systematic review of neuromarketing perspectives across consumer buying stages
Source: Front Neuroergon. 2025 Jul 11;6:1542847. doi: 10.3389/fnrgo.2025.1542847 (PMC12305819; doi:10.3389/fnrgo.2025.1542847)
Supplement: Supplementary file 1 [file Data_Sheet_1.docx]

Table 1: Keyword Arrays Metadata

| **Project Name: Neuromarketing and consumer neuroscience**  **Project Type: Systematic Literature Review**  **Owner: Authors**  **Start Date:** | | | |
| --- | --- | --- | --- |
| **Search Date** | **Database** | **Keywords** | **Search Results** |
| 14^th^ April, 2025  13:02 PM | EBSCO Host  Business Source Premier  EConLit with Full text | ( "consumer neuroscience" OR "consumer neuro-science" OR "consumer neuro science" OR "neuromarketing" OR "neuro-marketing" OR "neuro marketing" ) AND ( "consumer buying stag*" OR "buying decision*" OR "buying process*" OR "purchase decision*" OR "consumer decision*" OR "consumer purchas* journey*" OR "purchas* journey*" OR "purchas* decision*") | 844 Results  Time Period (2020-2025) – 437  Academic journal in English language – 246 |
| 14^th^ April, 2025  10:30 AM | Scopus in articles, Keywords and title | ( "consumer neuroscience" OR "consumer neuro-science" OR "consumer neuro science" OR "neuromarketing" OR "neuro-marketing" OR "neuro marketing" ) AND ( "consumer buying stag*" OR "buying decision*" OR "buying process*" OR "purchase decision*" OR "consumer decision*" OR "consumer purchas* journey*" OR "purchas* journey*" OR "purchas* decision*") | 1541 Results  Time period (2020-2025)– 1240  Journal Articles and review in English language– 740  Limit to Subject area "Business, Management & Accounting" & “Engineering” & “Social Sciences” – 403 |
| 14^h^ April, 2025  10:40 AM | Web of Science | ( "consumer neuroscience" OR "consumer neuro-science" OR "consumer neuro science" OR "neuromarketing" OR "neuro-marketing" OR "neuro marketing" ) AND ( "consumer buying stag*" OR "buying decision*" OR "buying process*" OR "purchase decision*" OR "consumer decision*" OR "consumer purchas* journey*" OR "purchas* journey*" OR "purchas* decision*") | 818 Results  Time period (2020-2025)– 660  Limit to document types -articles, review articles, early access in English – 583  WoS Categories – Business, Management & Engineering – 236 |

Table 2: Inclusion and exclusion criteria

| Description | Reason for inclusion | Reason for exclusion | Exemplary evidence |
| --- | --- | --- | --- |
| Time-period | Articles published between 2020 and 2025 to ensure the inclusion of all relevant contemporary research. | Not applicable |  |
| Conceptual boundaries | Use of interpretive approach to capture the diverse interpretations of the term neuromarketing and consumer neuroscience by different authors. | Not applicable | (Plassmann et al., 2012);(Hubert & Kenning, 2008) |
| Search terms | Boolean logic with regard to neuromarketing and consumer neuroscience in business and management, Engineering and Social Science narrows down the number of articles to those that make use of the relevant key terms. | Not applicable | Müller-Seitz (2012);(Mishra et al., 2021) |
| Database | Include interdisciplinary research by exploring a wide range of databases, including EBSCO Host  Business Source Premier  EConLit with Full text, Scopus, Web of Science | Not applicable | Bititci *et al*. (2012);(Goyal & Kumar, 2021) |
| Quality criteria | - ABS ranked 3/ 4/ 4* journal articles - ABDC ranked A/A* journal article - Scimago ranked Q1 journal articles - Empirical and theoretical articles to capture the full spectrum of work on this topic. | - ABS 1/ 2 journals - ABDC B/C/D journals - Scimago Q2/Q3/Q4 journals - JCR Q4 journals - All non-scholarly peer-reviewed articles, books, and non-published materials | Baldacchino *et al*. (2015); Pilbeam *et al*. (2012);(Jebarajakirthy et al., 2021) |
| Fit-for-purpose criteria | (1) Indicates aspects of marketing, business or management linked to consumer neuroscience or neuromarketing (2) context concerns consumer decision making, (3) linked to stages of purchase, and/or (4) uses attitudinal aspects | The papers not focussing on business, management, engineering, social science domain and not from the marketing areas. | Adams *et al*. (2016);  Boaz and Ashby (2003);(Christenson et al., 2017) |
